# Sudden death with pseudomembranous fibrinonecrotic enteritis in a zoo-housed hippopotamus (*Hippopotamus amphibius*): predominant isolation and genomic characterization of *Clostridium perfringens* type A

**DOI:** 10.4142/jvs.26036

**Published:** 2026-07-14

**Authors:** Thach Phan Van, Byungkwan Oh, Hadeer Khaled, Hyun Jong Park, Jaime C. Cabarles, Chae Woong Lim, Bumseok Kim, John Hwa Lee, Sang-Ik Oh

**Affiliations:** 1 Laboratory of Veterinary Pathology and Biosafety Research Institute, College of Veterinary Medicine, Jeonbuk National University, Iksan 54596, Korea.; 2 Faculty of Applied Science and Technology (FAST), Nguyen Tat Thanh University, Ho Chi Minh City 700000, Vietnam.; 3 College of Agriculture, Resources and Environmental Science, Central Philippine University, Jaro, Iloilo City 5000, Philippines.; 4 Department of Public Health, College of Veterinary Medicine, Jeonbuk National University, Iksan 54596, Korea.

**Keywords:** Case report, *Clostridium perfringens* type A, *Hippopotamus amphibius*, death, sudden, whole-genome sequencing

## Abstract

**Importance:**

*Clostridium perfringens* type A (CPA) is a common intestinal commensal, but it has been implicated in peracute fatal enteritis under predisposing conditions. Although clostridial enteric disease is well characterized in domestic species, reports in captive megaherbivores are rare. In addition, genomic data for CPA isolates from zoo-housed wildlife remain scarce, limiting comparative epidemiologic investigations.

**Case presentation:**

A 40-year-old female zoo-housed hippopotamus (*Hippopotamus amphibius*) was found dead without prior clinical signs. Necropsy revealed diffuse pseudomembranous fibrinonecrotic and hemorrhagic enteritis. Histopathology showed acute mucosal necrosis with an overlying fibrinonecrotic pseudomembrane and abundant intralesional bacilli. Aerobic culture yielded *Escherichia coli* without major virulence genes, whereas anaerobic culture yielded abundant β-hemolytic colonies. Multiplex toxinotyping polymerase chain reaction identified the predominant anaerobe as CPA (*plc*-positive only). Whole-genome sequencing yielded a 3.25-Mb draft assembly comprising 37 putative chromosomal contigs (3.20 Mb total) and one 48-kb putative plasmid contig. *In silico* screening detected *plc*, *pfoA*, *colA*, *cloSI*, multiple sialidase/hexosaminidase genes, and predicted antimicrobial resistance determinants [*mprF* and *tetA(P*)/*tetB(P)*].

**Conclusions and Relevance:**

To our knowledge, this is the first genomic characterization of a CPA isolate from an adult hippopotamus, providing a reference for zoo-based surveillance and outbreak investigations. Clostridial-type fibrinonecrotic–hemorrhagic enteritis should be considered in the differential diagnosis of sudden death in adult zoo-housed hippopotami.

## INTRODUCTION

*Clostridium perfringens* is a Gram-positive, spore-forming anaerobic bacterium that causes a wide spectrum of diseases in humans and animals, ranging from gas gangrene to fatal enterotoxemias [1]. Based on six major typing toxin genes, *C. perfringens* is currently divided into seven toxinotypes (A–G), of which type A (CPA) strains are the most prevalent and clinically important [2,3,4]. Toxinotype A strains—defined by the presence of the alpha-toxin gene (*plc*) and the absence of the other major typing toxin genes—have been reported in association with necrotic or hemorrhagic enteritis and enterotoxemia in domestic ruminants, pigs, foals, and occasionally wildlife [3,5].

Although *C. perfringens*–associated enteric disease is well documented in humans and domestic animals [5], clinicopathologic descriptions in large megaherbivores remain limited, with only sporadic reports in hippopotamids, rhinoceroses, and elephants [6,7]. The hippopotamus is a large semi-aquatic herbivore and a non-ruminating foregut fermenter with a complex forestomach, which may influence gastrointestinal microbial ecology [8]. As the species is widely maintained in zoological collections, husbandry practices (e.g., diet management, enclosure changes, and routine procedures) are critical determinants of gastrointestinal stability and may predispose to opportunistic enteric disease under disruption of gut homeostasis.

In zoological collections, sudden death events in large captive herbivores pose substantial welfare and biosecurity challenges, and rapid, practical diagnostic workflows are essential. Because CPA can be detected in clinically normal mammals, etiologic attribution in single cases requires careful integration of lesion patterns and bacteriology and, when feasible, toxin detection and/or *in situ* confirmation. Fatal clostridial infections in megaherbivores are rarely documented, and only a single neonatal case of CPA-associated enterotoxemia has been reported in a captive hippopotamus from South Korea [7]. Here, we describe peracute fatal enteritis in an adult zoo-housed hippopotamus in which CPA was the predominant β-hemolytic anaerobic isolate, and we provide a genome-based reference to support future comparative and epidemiologic investigations.

## CASE PRESENTATION

A 40-year-old female hippopotamus (*Hippopotamus amphibius*) housed at Jeonju Zoo (Republic of Korea) was found dead without preceding clinical signs. The animal had been maintained under standard zoo husbandry conditions and fed grass hay, pelleted herbivore feed, and seasonal vegetables. No recent dietary changes, medical treatments, or environmental stressors were recorded in the preceding weeks. The only notable environmental condition was hot summer weather around the time of death, with a recorded daily maximum ambient temperature of 34°C. The animal was last observed clinically normal at approximately midnight, and the carcass was discovered in the early morning. Necropsy commenced approximately 4 h after discovery.

Grossly, the skeletal muscles were diffusely pale pink. In the intestinal tract, the mucosal surface was diffusely covered by adherent fibrinous pseudomembranes and showed marked hyperemia (Fig. 1A and B). Minimal content was present in the small intestine, with no gas distension throughout the gastrointestinal tract. Microscopically, the small intestine showed severe villous necrosis and acute mucosal blunting with an overlying fibrinonecrotic pseudomembrane and mucous exudate (Fig. 1C). Marked mucosal hemorrhage and submucosal congestion were also present (Fig. 1D). Severe inflammatory cell infiltration, composed predominantly of neutrophils with admixed mononuclear cells, was observed mainly within the mucosa (Fig. 1E). Notably, numerous intralesional bacilli were present within the necrotic/exudative mucosa (Fig. 1F), which were confirmed as Gram-positive bacilli consistent with *C. perfringens* morphology by gram staining. No significant lesions were observed in other major organs.

**Fig. 1:**
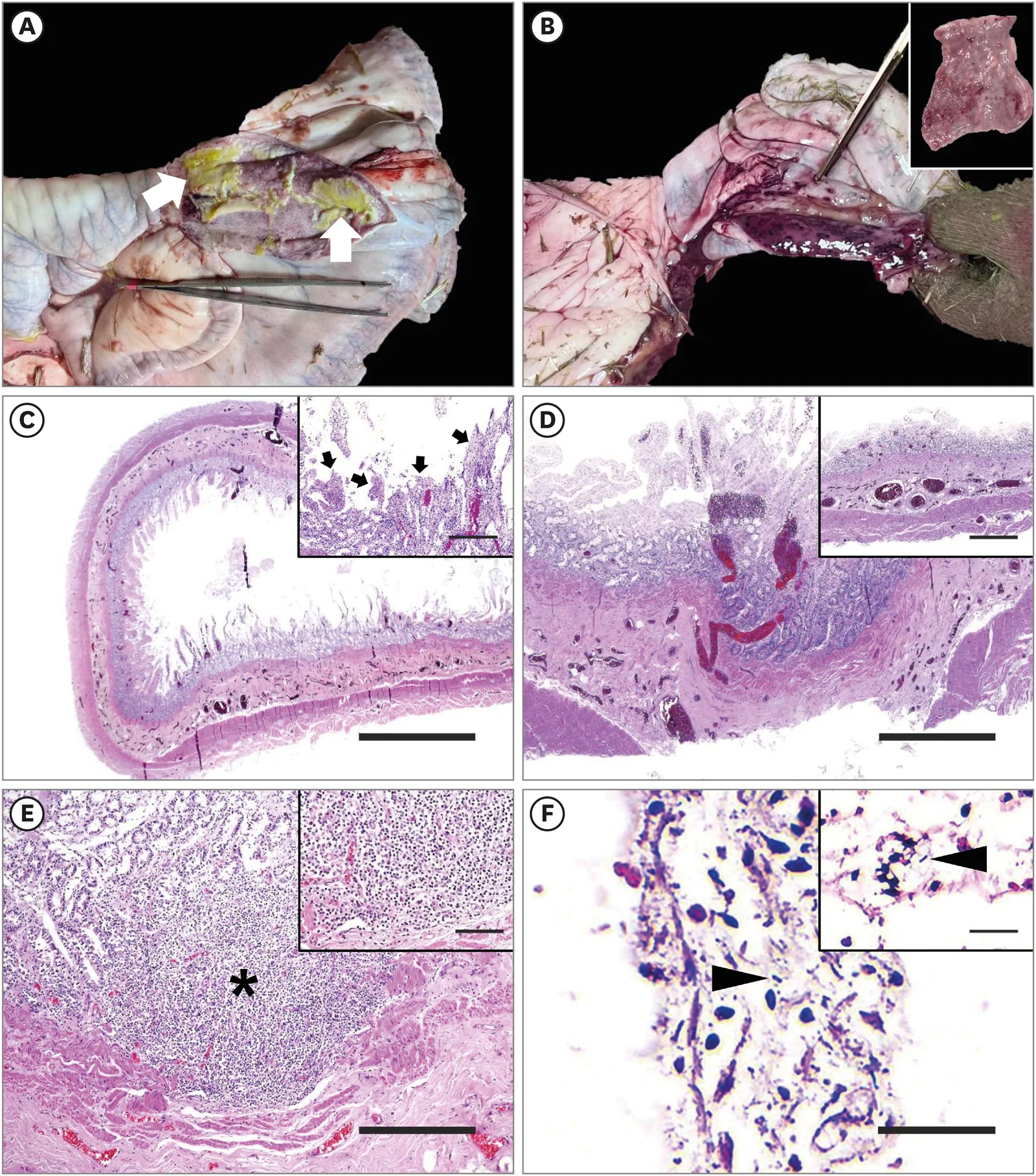
Gross and histopathological lesions in a 40-year-old female hippopotamus (*Hippopotamus amphibius*). (A) Small intestine showing diffuse, adherent fibrinous pseudomembranes (white arrows). (B) Longitudinally opened intestinal segment containing dark-red hemorrhagic luminal contents with marked mucosal congestion and hemorrhage; inset shows the mucosal surface after removal of luminal contents. (C) Small intestine. Severe villous necrosis and acute mucosal blunting with a fibrinonecrotic pseudomembrane and mucous exudate overlying villus tips; scale bar: 1,600 μm. Inset highlights superficial necrosis and fibrinonecrotic pseudomembranes with associated exudate (black arrows); scale bar: 200 μm. H&E. (D) Marked mucosal hemorrhage with prominent submucosal congestion in small intestine; scale bar: 500 μm. Inset shows severe submucosal congestion; scale bar: 500 μm. H&E. (E) Severe infiltration of neutrophils and mononuclear cells (asterisk) within the mucosa; scale bar: 200 μm. Inset highlights neutrophil and mononuclear cell infiltration at higher magnification; scale bar: 50 μm. H&E. (F) Numerous intralesional bacilli within necrotic/exudative mucosa (arrowhead); scale bar: 20 μm. H&E. Inset demonstrates numerous large gram-positive bacilli within necrotic/exudative mucosa (arrowhead); scale bar: 20 μm. Gram stain. H&E, hematoxylin and eosin.

Intestinal contents and small intestinal tissue were submitted for bacteriological examination. Aerobic culture yielded *Escherichia coli* without major virulence genes, whereas anaerobic culture on blood agar plates incubated at 37°C under anaerobic conditions yielded abundant growth of large β-hemolytic colonies. Genomic DNA was extracted from isolated β-hemolytic colonies using a commercial kit (QIAamp DNA Mini Kit; Qiagen, Germany). Multiplex polymerase chain reaction (PCR) targeting the major *C. perfringens* toxin genes was performed as previously described [2]. The isolate was positive only for the plc gene and negative for the other five major toxinotyping genes, consistent with CPA.

To further characterize the predominant anaerobic isolate, whole-genome sequencing (WGS) was performed using an Illumina paired-end platform. *De novo* assembly yielded a draft genome (38 contigs; total 3,249,720 bp; GC 28.1%), including chromosomal contigs totaling 3,201,590 bp and a 48,130 bp contig predicted to represent a plasmid (GC 27.3%). The genome was annotated using Prokka [9], identifying 2,871 predicted coding sequences, ribosomal RNA genes (5S, 16S, and 23S), 79 transfer RNAs, and a single transfer-messenger RNA (Fig. 2A). The draft genome assembly of OH-TH01 strain was deposited in GenBank (National Center for Biotechnology Information) under accession number JBTWEQ000000000 (BioProject PRJNA1404688). Comparative analysis with 28 reference *C. perfringens* genomes placed the isolate within lineage V, clustering most closely with strain EHE-NE18 in the phylogenetic reconstruction (Fig. 2B, Table 1), supporting lineage-level placement [10].

**Fig. 2:**
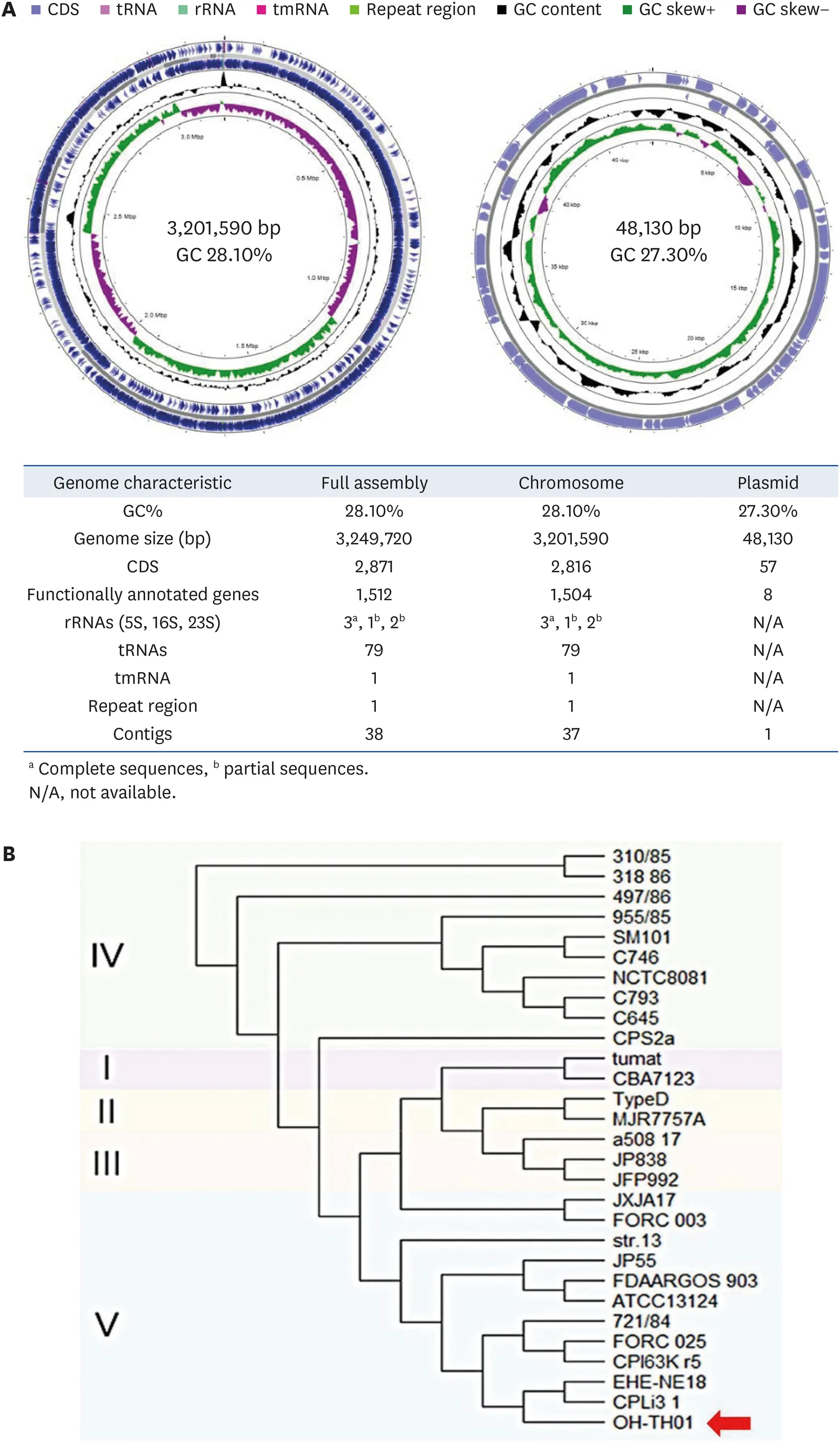
Genome atlas and phylogenetic analysis of the *Clostridium perfringens* isolate (OH-TH01) from a hippopotamus. (A) Circular map of the draft genome assembly showing the chromosomal contig (left) and a putative plasmid contig (right). From outer to inner, rings represent: CDS on the forward and reverse strands (blue), tRNA (light purple), rRNA (light green), tmRNA (pink), GC content (black), GC skew (positive, green; negative, purple), and the scale. The genome atlas was generated using Proksee via the CGView server (https://proksee.ca/). (B) Phylogenetic tree of OH-TH01 with 28 reference *C. perfringens* genomes, illustrating clustering into major lineages (I–V). The hippopotamus isolate (OH-TH01) is indicated by a red arrow and clusters within lineage V. CDS, coding sequence; tRNA, transfer RNA; rRNA, ribosomal RNA; tmRNA, transfer-messenger RNA; N/A, not available.

**Table 1:** Reference genomes of *Clostridium perfringens* strains used for phylogenetic tree analysis in this study

Assembly name	NCBI assembly accession	Strain	Lineage	Host/source	Isolation year	Isolation country
ASM399026v1	GCA_003990265.1	tumat	I	Canine	2012	Russia
ASM235579v1	GCF_002355795.1	CBA7123	I	Human	N/A	South Korea
ASM638542v1	GCA_006385425.1	Type D	II	Sheep	2016	India
ASM154635v1	GCF_001546355.1	MJR7757A	II	Human	N/A	N/A
ASM194979v1	GCF_001949795.1	JFP992	III	Equine	N/A	USA
cp508.17	GCF_900604525.1	a508.17	III	Cattle	N/A	France
ASM157978v1	GCF_001579785.1	JP838	III	Canine	2009	USA
ASM2013833v1	GCA_020138335.1	CPS 2a	IV	Swine	N/A	N/A
48269_C01	GCF_900461295.1	NCTC8081	IV	Human	1946/1948	Germany
ASM1384v2	GCF_000013845.2	SM101	IV	Food poisoning	1953	N/A
ASM2013861v1	GCA_020138615.1	C645	IV	Food poisoning	2003	Finland
ASM2013853v1	GCA_020138535.1	955/85	IV	Food poisoning	1985	Germany
ASM2013855v1	GCA_020138555.1	C793	IV	Food poisoning	2005	Finland
ASM2013857v1	GCA_020138575.1	C746	IV	Food poisoning	2004	Finland
ASM2013863v1	GCA_020138635.1	497/86	IV	Food poisoning	1986	Germany
ASM2013867v1	GCA_020138675.1	318/86	IV	Food poisoning	1986	Germany
ASM2013869v1	GCA_020138695.1	310/85	IV	Food poisoning	1985	Germany
ASM1602737v1	GCF_016027375.1	FDAARGOS_903	V	N/A	N/A	N/A
ASM968v1	GCF_000009685.1	str. 13	V	Soil	N/A	N/A
ASM335094v1	GCA_003350945.1	JXJA17	V	Porcine	2017	China
ASM130473v1	GCF_001304735.1	FORC_003	V	Environment	2014	South Korea
ASM157976v1	GCF_001579765.1	JP55	V	Equine	2007	Canada
ASM1328v1	GCF_000013285.1	ATCC 13124	V	N/A	N/A	N/A
ASM320345v1	GCA_003203455.1	EHE-NE18	V	*Gallus gallus*	2002	Australia
ASM2013841v1	GCA_020138415.1	CPLi3-1	V	Environment	2003	Finland
ASM2013859v1	GCA_020138595.1	721/84	V	Food poisoning	1984	Germany
ASM185408v1	GCA_001854085.1	FORC_025	V	Human	2015	South Korea
ASM2013845v1	GCA_020138455.1	CPI 63K-r5	V	Human	2003	Finland

N/A, not available.

Genomic screening for putative virulence and antimicrobial resistance determinants was performed to support comparative interpretation of the isolate. The draft genome was screened against the Virulence Factor Database and Comprehensive Antibiotic Resistance Database databases [11,12]. Putative virulence-associated genes included *plc* (alpha-toxin), *pfoA* (perfringolysin O), *colA* (collagenase), *cloSI* (alpha-clostripain), and multiple predicted sialidase/hexosaminidase genes (*nanH*, *nanI*, *nanJ*, *nagH*, *nagI*, *nagJ*, and *nagK*) (Table 2). Predicted antimicrobial resistance determinants included *mprF* and tetracycline-associated genes *tetA(P)*/*tetB(P)*.

**Table 2:** Virulence-associated genes and antimicrobial resistance determinants identified in *Clostridium perfringens* isolate OH-TH01 from a hippopotamus

Category	Target gene	Predicted product/function	Method	Result
Major typing toxin genes	*plc*	Alpha-toxin	Multiplex PCR; WGS	+
Major typing toxin genes	*cpb*	Beta-toxin	Multiplex PCR; WGS	−
Major typing toxin genes	*etx*	Epsilon-toxin	Multiplex PCR; WGS	−
Major typing toxin genes	*iap*	Iota-toxin	Multiplex PCR; WGS	−
Major typing toxin genes	*cpe*	Enterotoxin	Multiplex PCR; WGS	−
Major typing toxin genes	*netB*	NetB toxin	Multiplex PCR; WGS	−
Additional toxins/enzymes	*pfoA*	Perfringolysin O	WGS	+
Additional toxins/enzymes	*colA*	Collagenase (kappa toxin)	WGS	+
Additional toxins/enzymes	*cloSI*	Clostripain	WGS	+
Additional toxins/enzymes	*cpb2*	Beta2 toxin	WGS	−
Sialidases	*nanH*, *nanI*, *nanJ*	Sialidases	WGS	+
Hexosaminidases	*nagH*, *nagI*, *nagJ*, *nagK*	Hexosaminidases	WGS	+
Antimicrobial resistance	*mprF*	Multiple peptide resistance factor	WGS	+
Antimicrobial resistance	*tetA(P)*	MFS antibiotic efflux pump	WGS	+
Antimicrobial resistance	*tetB(P)*	Tetracycline-resistant ribosomal protection protein	WGS	+

Gene presence/absence by WGS was determined with ≥ 96.68% identity and ≥ 99.97% coverage thresholds. ‘−’ indicates no hit above threshold.

PCR, polymerase chain reaction; WGS, whole-genome sequencing.

## DISCUSSION

CPA is ubiquitous in the environment and gastrointestinal tract of animals, yet it can cause peracute, often fatal enteric disease under predisposing conditions [5]. In the present case, an adult hippopotamus was found dead without premonitory signs and showed severe fibrinonecrotic and hemorrhagic enteritis with abundant intralesional bacilli within necrotic/exudative mucosa. The lesion pattern was compatible with clostridial-type enteritis described in domestic species.

Bacteriological examination supported a clostridial-type process in that anaerobic culture yielded abundant growth of large β-hemolytic colonies, whereas aerobic culture yielded *E. coli* only. Multiplex toxinotyping PCR identified the predominant anaerobic isolate as CPA. Because *C. perfringens* may be present as an enteric commensal, etiologic attribution in single cases is challenging [13]. Differential diagnoses for acute hemorrhagic enteritis in large herbivores include salmonellosis, other clostridial enteric diseases, ischemic or obstructive intestinal injury, and septicemic bacterial disease. In this case, *Salmonella* spp. was not detected, and other bacterial pathogens were not isolated from samples. Therefore, the combination of compatible gross and histopathologic lesions, abundant intralesional bacilli on hematoxylin and eosin (H&E), and predominant isolation of a β-hemolytic anaerobe with a toxinotype A profile supported a presumptive diagnosis of clostridial-type fibrinonecrotic/hemorrhagic enteritis with predominant isolation of CPA. Compared with the only previous report of CPA-associated disease in a neonatal hippopotamus [7], which described hemorrhagic enteritis with mucoid and hemorrhagic changes on the mucosal surface, the present adult hippopotamus showed a similar pathological lesion pattern, including fibrinonecrotic pseudomembrane formation and marked intestinal hyperemia. Although etiologic confirmation remains presumptive in the absence of toxin detection or *in situ* confirmation, these findings indicate that adult *H. amphibius* may develop a comparable clostridial-type enteritis associated with predominant CPA isolation.

A specific predisposing trigger could not be identified in this case because no recent dietary change, antimicrobial administration, medical treatment, or husbandry procedure was recorded. The only notable environmental condition was hot summer weather around the time of death, with a recorded daily maximum ambient temperature of 34°C. Although high ambient temperature may have acted as a nonspecific environmental stressor, there was no direct evidence of heat stress, dehydration, or heat-associated intestinal dysbiosis; therefore, this factor should be interpreted cautiously.

WGS of the predominant isolate provided additional insight into genomic features and a predicted virulence repertoire. The overall genome characteristics were consistent with published *C. perfringens* genomes [14]. The predicted virulence profiles included membrane-damaging toxins (*plc* and *pfoA*), tissue-degrading enzymes (*colA* and *cloSI*), and mucosal barrier-modifying sialidases/hexosaminidases (*nag*/*nan* family members), all of which have been implicated in severe clostridial enteric disease (Table 2) [15,16,17,18]. Phylogenomic analysis clustered OH-TH01 within lineage V and most closely with strain EHE-NE18 [19], supporting the broad host distribution of related *C. perfringens* lineages. The genome also harbored predicted antimicrobial resistance determinants, including *mprF* and *tetA(P)*/*tetB(P)* [20]. Because these findings were based on *in silico* screening without phenotypic antimicrobial susceptibility testing, they should be interpreted as predicted determinants rather than confirmed clinical resistance. Nonetheless, these results highlight the value of continued antimicrobial stewardship and surveillance in captive settings where resistant anaerobes may emerge or circulate.

Key limitations include the absence of direct toxin detection in intestinal contents, lack of *in situ* confirmation of the organism within lesions (e.g., IHC/FISH), and lack of phenotypic antimicrobial susceptibility testing. Nonetheless, this report expands the clinicopathologic spectrum of clostridial-type enteritis in captive megaherbivores. To the best of our knowledge, the present case report also provides the first genomic characterization of a CPA isolate from an adult hippopotamus, offering a reference for zoo-based surveillance and comparative outbreak investigations.

In conclusion, clostridial-type fibrinonecrotic and hemorrhagic enteritis should be considered in the differential diagnosis of sudden death in adult hippopotami housed in zoological collections. In practice, a presumptive diagnosis can be supported by prompt necropsy with histopathology and anaerobic culture, together with toxinotyping PCR to characterize predominant *C. perfringens* isolates. In this case, the concordance of lesion pattern, abundant intralesional bacilli on H&E, and predominant isolation of CPA supported a presumptive diagnosis of clostridial-type fibrinonecrotic-hemorrhagic enteritis with predominant isolation of CPA, while definitive etiologic confirmation would require toxin detection and/or *in situ* demonstration of the organism within lesions. WGS of OH-TH01 strain provided additional genomic context and a reference dataset for future comparative and epidemiologic investigations in captive megaherbivores.
